# Microbiota Implications in Endocrine-Related Diseases: From Development to Novel Therapeutic Approaches

**DOI:** 10.3390/biomedicines12010221

**Published:** 2024-01-18

**Authors:** Vicente Javier Clemente-Suárez, Laura Redondo-Flórez, Alejandro Rubio-Zarapuz, Alexandra Martín-Rodríguez, José Francisco Tornero-Aguilera

**Affiliations:** 1 Faculty of Sports Sciences, Universidad Europea de Madrid, Tajo Street, s/n, 28670 Madrid, Spain; vctxente@yahoo.es (V.J.C.-S.); alejandro.rubio.z@hotmail.com (A.R.-Z.); josefranciso.tornero@universidadeuropea.es (J.F.T.-A.); 2 Grupo de Investigación en Cultura, Educación y Sociedad, Universidad de la Costa, Barranquilla 080002, Colombia; 3 Department of Health Sciences, Faculty of Biomedical and Health Sciences, Universidad Europea de Madrid, C/ Tajo s/n, 28670 Villaviciosa de Odón, Spain; lauraredondo_1@hotmail.com

**Keywords:** microbiota, endocrine-related cancers, hormones, endocrine pathology, dysbiosis, gut microbiome, endocrine disorders

## Abstract

This comprehensive review article delves into the critical role of the human microbiota in the development and management of endocrine-related diseases. We explore the complex interactions between the microbiota and the endocrine system, emphasizing the implications of microbiota dysbiosis for the onset and progression of various endocrine disorders. The review aims to synthesize current knowledge, highlighting recent advancements and the potential of novel therapeutic approaches targeting microbiota-endocrine interactions. Key topics include the impact of microbiota on hormone regulation, its role in endocrine pathologies, and the promising avenues of microbiota modulation through diet, probiotics, prebiotics, and fecal microbiota transplantation. We underscore the importance of this research in advancing personalized medicine, offering insights for more tailored and effective treatments for endocrine-related diseases.

## 1. Introduction to the Human Microbiota and Its Composition

The human microbiota, an intricate and dynamic consortium of microorganisms, plays a pivotal role in human health and disease. This community, primarily composed of bacteria along with fungi, viruses, and archaea, is predominantly harbored in the gut but also resides in various bodily habitats like the skin, oral cavity, respiratory tract, and urogenital tract. Each of these ecosystems hosts a unique microbial community, reflective of its specific environmental conditions and host interactions [1].

The gut microbiota, the most extensively studied, is impressively diverse, housing approximately 100 trillion microbial cells, a number roughly equivalent to the human body’s cell count [2]. This microbial diversity is not just vast in number but also in the variety of species, with estimates suggesting the presence of over a thousand bacterial species, although a few dominant phyla like Firmicutes and Bacteroidetes comprise the majority [3]. Its composition is shaped by numerous factors, including genetics, age, diet, environment, and lifestyle. The initial colonization of the microbiota in newborns is influenced significantly by the mode of delivery and early feeding practices [4]. Vaginally delivered infants typically acquire bacterial communities resembling their mother’s vaginal microbiota, predominantly *Lactobacillus* and *Prevotella*, while cesarean-section delivered infants are more likely to have a microbiota resembling skin flora, with higher proportions of Staphylococcus and Corynebacterium [5].

As the individual matures, the microbiota diversifies, reflecting dietary changes and environmental exposures [6]. Diet has a profound impact, with dietary fibers fostering a microbiota rich in short-chain fatty acid (SCFA) producers, influencing both gut health and systemic immunity [6]. Furthermore, antibiotic usage and lifestyle factors like exercise can significantly alter the microbiota composition [7].

Then, a relationship exists between the human host and its microbiota that is largely symbiotic [8]. The microbiota aids in digestion, synthesizes essential vitamins, modulates the immune system, and provides a line of defense against pathogenic organisms [9]. Dysbiosis, a disruption in this delicate balance, has been implicated in a range of diseases, from gastrointestinal disorders to neurological conditions [10]. The gut-brain axis exemplifies the extent of microbiota-host interactions, where gut bacteria influence brain function and behavior, suggesting the microbiota’s role extends beyond the traditional boundaries of digestive health [11]. Recent studies have also highlighted the microbiota’s role in metabolizing drugs and influencing their efficacy and toxicity, marking its significance in precision medicine [12].

Advancements in sequencing technologies, particularly next-generation sequencing, have revolutionized our understanding of the human microbiota [12]. Techniques like 16S rRNA gene sequencing and whole-genome shotgun sequencing provide insights into the composition and functional potential of these microbial communities [13]. Metabolomic and transcriptomic analyses further complement these studies, offering a more comprehensive understanding of the microbiota’s functional roles [14]. These technological advancements have enabled researchers to elucidate the intricate relationships between the microbiota and various physiological processes [15]. For instance, metagenomic analyses have uncovered specific microbial genes and pathways involved in nutrient metabolism, resistance to pathogens, and immune system modulation [16]. This genetic and functional diversity underscores the microbiota’s adaptability and its critical role in maintaining host homeostasis [17].

Moreover, the human microbiota is not static but dynamically interacts with the host’s immune system [18]. The development of the immune system, particularly in early life, is profoundly influenced by microbial colonization [19]. Studies have shown that germ-free animals, which lack a microbiota, exhibit underdeveloped immune systems, indicating the microbiota’s essential role in immune maturation [20]. This symbiotic relationship extends to the maintenance of mucosal barriers and the regulation of inflammatory responses, both critical for preventing overreaction to non-pathogenic antigens [21].

The concept of the gut-brain axis, which posits a bidirectional communication pathway between the central nervous system and the gut microbiota, has garnered considerable interest [22]. This axis involves neural, hormonal, and immunological signaling mechanisms. For example, certain gut bacteria can produce neurotransmitters like serotonin and gamma-aminobutyric acid (GABA), which influence mood and behavior [23]. Conversely, stress and other brain-derived signals can affect gut permeability and microbiota composition, illustrating a complex interplay [24].

In addition to its role in maintaining health, the microbiota’s disruption, or dysbiosis, is increasingly implicated in a range of diseases [25]. For example, a shift in the gut microbiota’s composition has been associated with metabolic disorders such as obesity and diabetes. These changes can alter metabolic pathways, leading to increased energy harvest and inflammation, contributing to disease pathogenesis [25]. Furthermore, the role of the microbiota in drug metabolism is a burgeoning area of research [26]. The microbiota can directly metabolize drugs or modulate host drug metabolism pathways, affecting drug efficacy and toxicity [27]. This has significant implications for personalized medicine, as understanding individual microbiota compositions could guide more effective and safer drug therapies [28].

Thus, considering the complex and multifaceted interactions between the human microbiota and host physiology, particularly in the context of endocrine-related diseases, there is a compelling justification for the present narrative review. We aim to synthesize current knowledge and recent advancements in understanding the role of the microbiota in endocrine system development, maintenance, and dysfunction. Given the burgeoning evidence linking microbiota dysbiosis to various endocrine disorders, such as diabetes, obesity, and thyroid dysfunctions, a comprehensive examination of these interactions is timely and pertinent [29]. Additionally, exploring the potential of novel therapeutic approaches that target microbiota-endocrine interactions holds immense promise. These could include strategies like microbiota modulation through diet, probiotics, prebiotics, and fecal microbiota transplantation [30,31]. The review will not only consolidate existing research but also highlight gaps in knowledge and propose future research directions. Ultimately, it aims to contribute to the growing field of personalized medicine, where understanding an individual’s microbiota composition could lead to more tailored and effective treatments for endocrine-related diseases.

Thus, for the present narrative review, we adopted an exhaustive and systematic search strategy to compile relevant literature, following the methodology of previous authors [32,33,34]. Our search extended beyond conventional databases to include grey literature and expert consultations. Specifically, we utilized databases like PubMed, Scopus, Embase, Science Direct, Sports Discuss, ResearchGate, and the Web of Science and expanded to platforms such as Google Scholar for broader access to non-peer-reviewed material.

The literature search was meticulously designed, using MeSH-compliant keywords such as “human microbiota”, “endocrine system”, “microbiota-endocrine interactions”, “microbiota dysbiosis”, “hormone regulation”, “gut microbiome”, “endocrine disorders”, and “microbiota in drug metabolism”. This was to ensure comprehensive coverage of publications from 1 May 2003, to 1 May 2023, relevant to our review’s focus.

A team of nine experienced authors screened the titles and abstracts of all retrieved manuscripts, establishing inclusion criteria based on relevance, scientific rigor, and alignment with the review’s theme. Exclusion criteria were applied to manuscripts outside the specified timeline, not written in English, or not pertinent to our focused research area. This meticulous selection process was pivotal in ensuring the inclusion of high-quality, relevant studies.

The same team undertook the critical task of extracting and synthesizing data from the selected studies. Each study was independently reviewed, and its findings were incorporated into a cohesive narrative, ensuring a comprehensive and systematic review of the current knowledge in the field. This approach enabled us to present a balanced and thorough perspective on the intricate relationship between the human microbiota and endocrine-related diseases, highlighting developmental aspects and exploring novel therapeutic approaches.

## 2. Overview of the Endocrine System and Its Role in Maintaining Homeostasis

The endocrine system, an intricate and vital network within the human body, plays a crucial role in maintaining homeostasis—a condition of consistent internal physical and chemical equilibrium [35]. This system encompasses a series of specialized glands, including the pituitary, thyroid, adrenal, and pancreas [35]. Each of these glands secretes hormones directly into the bloodstream, which act as key chemical messengers [36]. These hormones orchestrate a multitude of essential bodily functions, enabling not only the survival of the organism but also its capacity to adapt to varying environmental conditions [37]. The regulation of these functions is critical, as underscored by previous research, which emphasizes the endocrine system’s central role in the integration and regulation of physiological processes [38].

Hormones produced by these endocrine glands are instrumental in overseeing various aspects of bodily function [39]. They play a decisive role in managing metabolism, the body’s conversion of food into energy, and subsequent growth and development [39]. These hormones are also vital in regulating mood and various reproductive processes, illustrating their influence over both physical and psychological aspects of human health [40]. The endocrine system’s functionality hinges on a series of intricate feedback mechanisms [41]. These mechanisms enable hormones to self-regulate their production based on the body’s needs, thereby maintaining a delicate balance of bodily functions [42]. Through feedback loops in relation to hormone synthesis, release, and action in relation to the body’s homeostatic needs [43].

The endocrine system’s intricate interactions with various bodily systems underscore its central role in maintaining overall physiological balance [37]. This interplay is particularly evident in its relationship with the immune system [44]. The immune system, which defends the body against pathogens, is profoundly influenced by endocrine activity, especially during stress responses and inflammatory processes [45]. Hormones like cortisol, produced by the adrenal glands, exhibit immunosuppressive effects, modulating the immune response during periods of stress [46]. Further on, cortisol and other glucocorticoids can alter leukocyte distribution, cytokine production, and ultimately the efficacy of the immune response. Indeed, it plays a pivotal role in the maintenance and regulation of the skeletal system [47]. Hormones such as parathyroid hormone (PTH) and vitamin D are crucial in regulating calcium and phosphate metabolism, which are fundamental to bone health and integrity [48]. PTH regulates calcium levels in the blood, while vitamin D ensures its absorption in the intestines [49]. The importance of this regulation is evidenced in conditions like osteoporosis, where hormonal imbalances disrupt bone remodeling, leading to decreased bone density and increased fracture risk [50].

Regarding energy balance and metabolism, the endocrine system exerts substantial influence through hormones like leptin, which is secreted by adipose tissue [51]. Leptin plays a critical role in regulating energy intake and expenditure by signaling the brain to adjust appetite and energy utilization [52]. This hormone is a key factor in the body’s energy homeostasis, and its dysregulation can lead to metabolic disorders, including obesity [53]. Further on, alterations in leptin signaling can disrupt metabolic balance and lead to increased fat storage.

Moreover, the endocrine system’s impact on mental health is increasingly recognized [54]. Hormones such as estrogen and testosterone are not only involved in reproductive functions but also play significant roles in regulating mood and cognitive functions [40]. Fluctuations in the levels of these hormones can influence the risk of developing mental health conditions such as depression and anxiety disorders [55]. On this line, hormonal changes across the lifespan can affect psychological well-being [56]. Their research suggests a link between hormone levels and the susceptibility to mood disorders, underscoring the endocrine system’s integral role in mental health [57]. Overall, the endocrine system’s interactions with the immune, skeletal, metabolic, and neurological systems illustrate its vital role in coordinating and maintaining bodily functions [58]. Understanding these complex interactions is essential for developing therapeutic approaches to manage various disorders related to hormonal imbalances and dysfunctions [59].

In conclusion, the endocrine system, a complex and essential network within the human body, plays a pivotal role in maintaining homeostasis and overall health [37]. It effectively integrates and coordinates various physiological processes through its intricate network of glands and hormones [60]. The system’s influence spans a wide array of bodily functions, from metabolism, growth, and development to stress response, immune function, and mental health. Its harmonious interaction with other systems underscores the holistic nature of bodily functions and the delicate balance required for optimal health [61]. As we continue to unravel the complexities of the endocrine system, its significance in both health and disease becomes increasingly apparent [39]. This understanding paves the way for innovative treatments and interventions for a range of endocrine-related disorders, highlighting the importance of continued research and exploration in this dynamic field of medicine [62]. The insights garnered from the study of the endocrine system not only enhance our understanding of human physiology but also provide a foundation for improving health outcomes across various medical disciplines [63].

## 3. The Emerging Concept of Microbiota-Endocrine System Interactions

### 3.1. Overview

An emerging area of scientific inquiry is the interplay between microbes and multicellular creatures. The influence of gut bacteria on the development of endocrine system disorders, including diabetes and thyroid illness, has been well-established [64]. Alterations in the composition, structure, and metabolites of the gut microbiota have been implicated in the development of gastrointestinal problems such as ulcers, intestinal perforation, and various inflammatory and autoimmune diseases [65]. There has been a growing body of literature in recent years that highlights the correlation between gut bacteria and endocrine system illnesses. The treatment centered around the gut flora has garnered significant attention in the interim [66].

**Figure 1:**
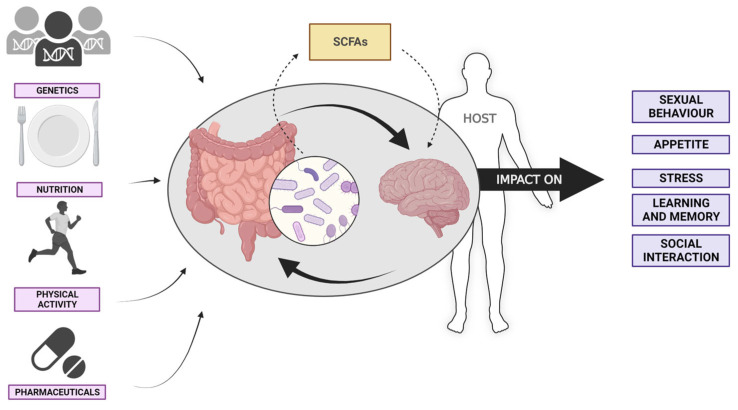
The gut’s health is influenced by genetics, physical activity, nutrition, and medications, which subsequently have implications for sexual behavior, hunger regulation, stress response, cognitive processes such as learning and memory, and social interaction.

The study conducted by Sender et al. (2016) provides evidence supporting the notion that the cellular structures of humans and bacteria exhibit a considerable degree of similarity [67]. The gut microbiome, which is sometimes referred to as the “second genome” of humans, stands out as the most intricate and prevalent category of flora within the bacterial systems present in the human body. The human gut microbiome undergoes alterations in its composition and structure throughout the course of time while maintaining a degree of dynamic stability [68]. Several factors, including nutrition, genetics, physical activity, and pharmaceutical interventions, have been identified as influential elements in shaping the composition of the gut microbiome (Figure 1) [69,70]. The maintenance of a balanced gut microbiome can be facilitated through the adoption of a proper diet and frequent exercise. Furthermore, it is important to include additional aspects that are often disregarded, such as the method of childbirth and infant feeding practices [71].

The gut microbiome confers numerous advantages to the human body, as evidenced by its impact on metabolic processes and immune function [72]. In recent times, there has been a significant surge in interest surrounding the correlation between disorders of the endocrine system and imbalances within the gut microbiota. The disruption of the intestinal epithelial cell barrier function, as well as alterations in specific metabolite levels, have been implicated in the pathogenesis of many endocrine disorders [73]. One example of these substances includes indigestible carbohydrates, such as cellulose, which can undergo metabolic processes in the distal intestine to produce short-chain fatty acids (SCFAs) (Figure 1) [74]. These carbohydrates have been found to be associated with various endocrine system disorders, including diabetes, chronic kidney disease (CKD), obesity, osteoporosis, and gout [75,76]. Furthermore, the dysbiosis of the gut microbiome has been implicated in the etiology of diabetes, thyroid illness, and autism, leading to detrimental pathogenic responses in the immune system [77,78]. Furthermore, it has been observed that the gut microbiome exerts an influence on the brain via the hypothalamic adrenal pituitary (HPA) axis in the pathophysiology of depression [79]. The gut microbiome is intricately linked to the brain through the hypothalamic-pituitary-adrenal (HPA) axis, a vital regulatory system that governs several physiological processes throughout the body (Figure 1) [80].

The process of bacterial colonization of the intestine has a significant influence on the development of the immune system and the endocrine system from the time of birth. The interaction between microorganisms and hormones has the potential to impact the metabolism, immune response, and behavior of the host organism. The bidirectional nature of this interplay is evident, as research has demonstrated that the microbiota is influenced by host hormones while also exerting an impact on them.

The topic of the microbial endocrinology study was initially defined by Lyte and Ernst in 1992. Their observations led them to conclude that the development of bacteria can be influenced by stress-induced neuroendocrine hormones. Additional investigations in the field of microbial endocrinology have revealed the presence of hormone receptors within microorganisms, leading to the hypothesis that these receptors serve as a means of intercellular communication [81]. The study conducted by Lyte and Bailey demonstrated that the presence of pathogenic neurotoxins, such as the neurotoxin 6-hydroxydopamine, can induce changes in norepinephrine levels in mice [82]. This finding highlights the reciprocal relationship between the host and the microorganisms involved in the interaction. According to a study with a focus on evolutionary aspects, it was shown that numerous enzymes responsible for the metabolism of hormones in host organisms, such as epinephrine, norepinephrine, dopamine, serotonin, and melatonin, could potentially have originated from horizontal gene transfer events involving bacteria [83].

The influence of host hormones on bacterial gene expression has been shown [84], and this interaction can subsequently impact the hosts. An illustration of this phenomenon can be seen in the way catecholamines augment the process of bacterial adherence to host tissues while also exerting an influence on the proliferation and pathogenicity of such bacteria [85]. For instance, in recent studies, it has been discovered that the intestinal microbiota plays a significant role in the regulation of bone metabolism by exerting its influence on host metabolism, immunological function, and hormone production [86]. The influence of bacteria on many host responses, such as behavior, metabolism, hunger, and immunological responses, is mediated via their endocrine actions.

### 3.2. Behavior and Emotional Regulation

The gut microbiota exerts an influence on the behavior of animals and humans through many mechanisms. According to Diaz et al. (2011) [87], germ-free mice exhibit modifications in cognitive function, memory, stress response, anxiety, and social behavior (Figure 1). The gut microbiota has the potential to impact human emotional states and disease conditions, including stress-related irritable bowel syndrome (IBS) and autism. These associations have been discussed in previous studies by Cryan and O’Mahony (2011) for IBS [88]. In recent studies, it has been demonstrated that gut bacteria have the capacity to both synthesize and react to neurohormones, including serotonin, dopamine, and norepinephrine. As an illustration, Salmonella exhibits a downregulation of its resistance to host antimicrobial peptides and stimulates crucial metal transport systems in response to host adrenaline. These mechanisms have a significant impact on the cellular balance of oxidative stress [89]. Also, more recently, it has been shown that oxidative stress and the generation of reactive oxygen species (ROS) provide a biological challenge to bacteria, prompting the activation of protein synthesis and the development of antioxidants as a defense mechanism. In this study, Huang et al. provide findings indicating that the glutamate dehydrogenase (GDH) enzyme in Salmonella can incorporate ammonium into glutamate, hence facilitating the production of glutathione (GSH) as a defense mechanism against oxidative stress [90].

The microbiome has the potential to contribute to emotional regulation and homeostasis through the modulation of stress hormone levels. Germ-free (GF) mice exhibit heightened levels of corticosterone and adrenocorticotropic hormone (ACTH) in their plasma when subjected to mild stress, as observed in studies conducted by Sudo et al. (2004) [91,92]. This physiological reaction is known to contribute to the manifestation of anxiety-related behaviors and stress-related symptoms in these animals. ACTH plays a crucial role in the functioning of the HPA axis by stimulating the synthesis and release of corticosteroids. In accordance with the findings of Messaoudi et al. (2011) [93], it has been observed that two species, namely *L. helveticus* and *B. longum*, can decrease the levels of cortisol, a stress hormone, as well as alleviate anxiety-like behavior in both rats and individuals who are in good health. In addition, the study conducted by Bravo et al. (2011) revealed that mice subjected to long-term administration of the probiotic *L. rhamnosus* had reduced levels of corticosterone and demonstrated less depressed behavior during a forced swim test as compared to the control group [94].

### 3.3. Sex Hormones

Instances of bacteria being influenced by sex hormones have been documented since the 1980s. As an example, it has been observed that *Prevotella intermedius* can uptake estrogen and progesterone, hence promoting its growth [95]. According to Menon et al. (2013), alterations in the expression of the estrogen receptor, specifically ER-β, have an impact on the composition of the gut microbiota [96]. This reciprocal relationship is evident since certain strains of bacteria have also been linked to the production or alteration of steroids [34]. An instance of this is the conversion of glucocorticoids to androgens, a collection of male steroid hormones, by *Clostridium scindens* [97]. The significance of intestinal bacteria in estrogen metabolism is noteworthy, as evidenced by the decrease in estrogen levels resulting from antibiotic usage [98]. Moreover, a significant association was seen between urinary estrogen concentrations and the diversity of the fecal microbiome [99]. Additionally, the presence of *Clostridia*, including non-*Clostridiales*, and three specific taxa within the *Ruminococcaceae* family exhibited high relationships with urine estrogen levels. Mittelstrass and colleagues proposed a potential interaction among the endocrine system, gut microbiota, and metabolism, which may be influenced by gender-specific variations in fatty acid profiles [100].

### 3.4. Appetite 

One of the fundamental functions of the gut microbiota is the breakdown and fermentation of diverse carbohydrates, resulting in the production of short-chain fatty acids (SCFAs). Germ-free (GF) mice exhibit distinct metabolic profiles compared to mice grown under normal conditions, characterized by reduced levels of SCFAs, hepatic triacylglycerol, and glucose. It is worth noting that the administration of subtherapeutic doses of antibiotics in mice, while not completely eradicating the gut microbial population, does result in substantial alterations to its composition. This, in turn, leads to elevated levels of SCFAs and subsequent weight gain [101]. The metabolic consequences of the microbiota can additionally impact the regulation of hormone synthesis derived from cholesterol, peptides, or amino acids. For example, previous studies have demonstrated that SCFAs can induce the secretion of serotonin (5-HT) and peptide YY (PYY), a hormone that is released following food intake and is associated with appetite suppression and reduced gastrointestinal motility [102]. The gut microbiota is believed to have an impact on many hormones, particularly neuropeptides, which play a crucial role in the regulation of hunger and metabolism. The aforementioned substances encompass alpha-melanocyte-stimulating hormone, neuropeptide Y, agouti-related protein, ghrelin, leptin, insulin, and other compounds [34]. A further consequence of bacterial influence on metabolic hormones may arise from the synthesis of somatostatin, a peptide hormone that inhibits the secretion of gastrointestinal and pancreatic hormones [103].

In summary, there is an increasing body of research that has established a connection between hormones and the microbiome in relation to immune responses in both healthy individuals and those with autoimmune diseasees. Numerous interrelationships exist between the microbiota and hormones, whereby they can exert their influence on the immune response via common pathways [104]. Hormones exert various effects on the immune system. The immune system and neuroendocrine system have a shared repertoire of hormones and receptors. Many advancements in this field have been achieved through the utilization of germ-free (GF) animals in experiments, as well as the implementation of probiotics (specific microbes believed to have beneficial effects on the host) and prebiotics (non-digestible carbohydrates that serve as nourishment for probiotics) [105]. These experimental approaches have been complemented by progress in sequencing and bioinformatics platforms. Although this area of study is still in its early stages, it is anticipated that the next research will uncover further significant correlations between hormones and the microbiome. The field of microbial endocrinology offers a potential explanation for the impact of the microbiota on the gastrointestinal (GI) and psychological well-being of the host. Thus, it is suggested that hormones play a crucial role in facilitating the interaction between hosts and microbes.

## 4. Microbiota Dysbiosis and Its Implications in Endocrine-Related Diseases

### 4.1. Overview

The gut microbiota has been identified as a significant contributor to the maintenance of the host’s nutritional, metabolic, and immunological equilibrium [106]. Furthermore, apart from its primary job of maintaining gastrointestinal homeostasis, the gastrointestinal system also plays a significant role in many metabolic processes, such as digestion and nutrition absorption, detoxification, and the synthesis of vitamins [107]. Moreover, the microbiota is also recognized as a significant contributor to the maturation and functioning of the lymphoid system, with approximately 70% of this system being located within the intestinal mucosa. Within the existing body of research, numerous instances have been documented wherein the intestinal microbiota displays a modified composition in several pathological conditions, such as obesity, as compared to the “physiological” microbiome. These examples encompass studies conducted on both mouse models and human subjects. Due to this rationale, a significant portion of the research conducted on the gut microbiota has been devoted to this subject matter [106].

**Figure 2:**
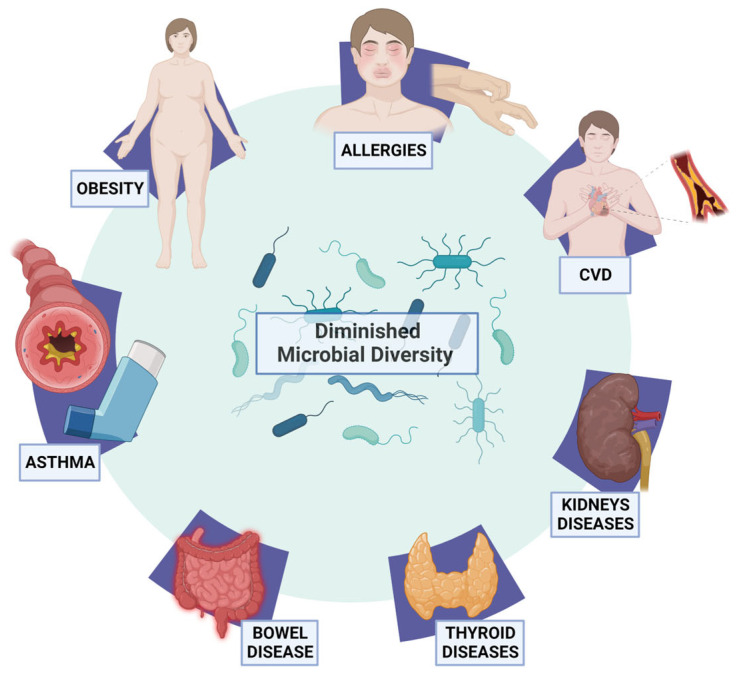
Possible diseases caused by low microbial diversity; cardiovascular disease (CVD).

Thus, the human microbiota appears to have a significant role in regulating both health and disease, and the importance of its impact on human health is increasingly becoming apparent. Gut dysbiosis refers to the disruption of the microbiota’s normal physiological function within the gastrointestinal tract. This condition can result in local inflammation and disturbances in metabolic processes [108]. Gut dysbiosis is characterized by a diminished microbial diversity [109], which has been associated with a wide array of human diseases (Figure 2). These include alterations in the immune status of the host as well as conditions such as asthma [110], allergies, inflammatory bowel disease [111], irritable bowel syndrome [112], obesity [113], thyroid disease [114], chronic kidney disease [33], cardiovascular disease (CVD) [115], and disruptions in blood pressure regulation [116].

Thus, there is evidence supporting a correlation between modified microbiota composition and the onset of various autoimmune disorders, hence indicating its involvement in the development of these diseases. In addition to those mentioned above, the conditions encompassed in this list are Type I diabetes [117], rheumatoid arthritis [118], systemic lupus erythematous [119], ulcerative colitis [120], atopic dermatitis [121], psoriasis vulgaris [122], and autoimmune neurological diseases [123]. Approximately 5–10% of the observed variation in bacterial taxonomy across people can be attributed to genetic factors. Most of the bacterial taxa that are transferred have been found to be associated with genes that play a role in innate immunity [124]. Given the significant global growth in the incidence of obesity, as well as the related illnesses of metabolic syndrome and type 2 diabetes (T2DM), it is imperative to direct our attention towards these topics in this review. Obesity is associated with an elevated susceptibility to certain diseases, including atherosclerosis, non-alcoholic fatty liver disease, and specific types of cancer. Emerging research indicates that the composition of the gut microbiota may have significant implications for the development and progression of obesity and its associated disorders [125]. Moreover, an increasing body of research suggests that the gut microbiota plays a pivotal role in the development and progression of thyroid diseases [126].

### 4.2. Diabetes and Obesity

Diabetes mellitus and obesity are two prominent global health concerns in the present century, characterized by substantial comorbidities and healthcare expenditures. The formation and progression of diabetes mellitus (DM) and obesity are influenced by various factors. However, recent research has shown that the microorganisms residing in the human gut may have significant involvement in these processes. Hu et al.’s recent study reported that there is a correlation between an unfavorable modification in the composition of the gut microbiome and increased insulin resistance, prolonged duration of diabetes, and the use of pharmaceuticals among individuals with diabetes [127]. The aforementioned attributes pertaining to diabetes were additionally linked to diminished quantities of specific butyrate-producing microorganisms that are responsible for generating short-chain fatty acids that contribute to overall well-being [128]. Other studies have also indicated that imbalances in the relative abundance of the gut microbiota may play a role in the development of weight gain and insulin resistance [129]. These imbalances include changes in the populations of Gammaproteobacteria and Verrucomicrobia, as well as alterations in the ratios of Firmicutes to Bacteroidetes, which have been associated with weight gain. Additionally, potential modifications in butyrate-producing bacteria, such as Faecalibacterium prausnitzii, have been implicated in the context of diabetes mellitus [130]. Furthermore, research has demonstrated that methanogenic archaea have the potential to impact the host’s metabolism and lead to weight gain. Nevertheless, a significant portion of research endeavors focus on utilizing stool or colonic samples, which may not accurately reflect the metabolic activity occurring within the small intestine [130].

Additionally, the close relationship between gut dysbiosis and gut permeability is also closely linked to elevated levels of circulating lipopolysaccharide and reduced levels of butyrate. These factors have the potential to disrupt immune responses and systemic mitochondrial function. Anderson’s recent study provides evidence of this disruption in a comprehensive analysis of various datasets pertaining to the pathophysiology of type 1 diabetes mellitus (T1DM), with a specific focus on the significance of changes in the mitochondrial melatonergic pathway inside pancreatic β-cells, which play a crucial role in the development of mitochondrial dysfunction [131]. The inhibition of mitochondrial melatonin results in an increased vulnerability of pancreatic β-cells to oxidative stress and impaired mitophagy. In this regard, the incorporation of the mitochondrial melatonergic pathway within pancreatic β-cells and their interacting cells has the potential to unify various sets of information that were previously separate in the context of T1DM [131]. Regarding therapeutic approaches for the treatment of Increased Intestinal Permeability, the comparative analysis of plant-based and animal-based diets revealed disparities in bacterial compositions and imbalanced proportions of bacterial phyla [132]. These discrepancies were associated with enhanced beneficial bacterial profiles and decreased activation of inflammatory cytokines, ultimately resulting in improved insulin sensitivity. In preliminary pilot trials, the administration of prebiotics and probiotics showed a decrease in inflammatory indicators [133]. However, it did yield modest enhancements in insulin resistance. Although the observed benefits were not substantial enough to justify therapeutic intervention in individuals with diabetes [134], the impact of a Mediterranean diet (MD) intervention in obese and diabetes patients resulted in a decrease in blood cholesterol levels and induced various alterations in the individual’s microbiome and metabolome, which hold significance for future approaches aimed at enhancing metabolic well-being [135].

### 4.3. Thyroid Disorders

There is a growing body of evidence suggesting the presence of a robust connection between the thyroid and the gastrointestinal system [136]. The findings suggest the presence of a lesser-known yet significant association between gut microbiota and the interplay of the immune system and thyroid function. Moreover, there exists a greater incidence of the simultaneous occurrence of thyroid and gastrointestinal disorders, such as the coexistence of Hashimoto’s Thyroiditis/Graves’ Disease and Celiac Disease/Non-celiac wheat sensitivity [123]. The presence of dysbiosis is frequently observed in individuals with thyroid problems. One aspect to consider is that it modifies the immune response by facilitating the process of inflammation and diminishing immunological tolerance. Consequently, it impairs the integrity of the intestinal membrane and results in an elevation of intestinal permeability. This, in turn, not only leads to heightened exposure to antigens but also triggers localized inflammation. Conversely, it possesses the ability to directly influence levels of thyroid hormones by means of its intrinsic deiodinase activity and the suppression of thyroid-stimulating hormone (TSH). The gut microbiota exerts an influence on the absorption of essential minerals for thyroid function, such as iodine, selenium, zinc, and iron. All of these elements are required for the proper functioning of the thyroid gland, and a discernible association exists between thyroid dysfunction and perturbations in the levels of these minerals [137]. Nevertheless, there exists a limited body of research investigating the potential association between gut microbiota and thyroid illness. Recently, the notion of a “thyroid-gut-axis” has been introduced. It has been shown that the principal pathway for iodine uptake in humans involves absorption through the gastrointestinal system and subsequent transfer to the thyroid gland. In this process, the billions of bacteria residing in the gut play a crucial role in the regulation of iodine metabolism. The primary mechanism for iodine uptake is facilitated by the sodium/iodine symporter (NIS). There is speculation regarding the potential mechanism for lipopolysaccharides (LPS) and SCFAs generated by the gut microbiota to modify thyroid iodine metabolism through the modulation of NIS expression and activity [138]. Recent research provides evidence for the key mechanisms underlying the disruption of thyroid homeostasis caused by lipopolysaccharide (LPS) [139]. Also, the excessive growth of bacteria, in a reciprocal manner, contributes to the compromised neuromuscular functionality of the gastrointestinal system, hence intensifying persistent gastrointestinal symptoms in individuals with hypothyroidism [140]. Regarding the pathophysiology of autoimmune thyroid diseases (AITD), it is not entirely comprehended. The etiology of this condition is typically attributed to the interplay of multiple endogenous and exogenous variables, including genetic predisposition, environmental influences, and immunological dysregulation [141]. Despite the absence of empirical evidence clarifying the precise mechanism underlying the relationship between microbiota and thyroid autoimmunity, existing research indicates that microbiota and its metabolites have the potential to influence thyroid immunology directly or indirectly, hence potentially contributing to the development of AITD [142].

Regarding cancer progression, the microbiota and its metabolites play a significant role as endogenous variables that have the potential to impact the progression of different types of malignancies. Nevertheless, there have been a limited number of published studies pertaining to the gut microbiota of individuals diagnosed with thyroid cancer. The findings of a serum metabolomic analysis conducted on patients with distant metastases originating from thyroid cancer indicate that the interplay between food and gut bacteria may have a significant impact on the aggressiveness of tumors [54]. As the investigation of the gut microecology pertaining to thyroid illnesses advances, mounting data suggests that the gut microbiota plays a significant role as an environmental component that directly or indirectly impacts the development of thyroid diseases. In this regard, Yu and collaborators reported that patients with thyroid carcinoma exhibit notable alterations in their gut microbiota. These observations contribute to the understanding of the connection between the gut microbiota and the development of thyroid carcinoma [143].

In summary, the reciprocal relationship between the gastrointestinal microbiota and the host’s immune system is well-established. The immune system plays a crucial role in modulating the microbiome community by maintaining a balance between pro- and anti-inflammatory pathways. Simultaneously, the microbiome plays a significant role in the development of the immune system [144].

### 4.4. Estrogens-Related Conditions

The exploration of the gut microbiome’s role in endocrine disorders extends beyond its impact on metabolic and stress-related hormones to encompass a wider range of endocrine functions. The microbiome’s influence on sex hormones, particularly estrogen, is a critical area of investigation. The gut microbiota plays a role in the enterohepatic circulation of estrogens, where certain bacterial enzymes are involved in the deconjugation and reabsorption of estrogens, impacting their overall levels and activity in the body. This mechanism, as described by Fuhrman et al. (2014), suggests a significant interplay between gut bacteria and estrogen-related conditions, such as breast cancer and endometriosis [99]. In the realm of reproductive health, the gut microbiome’s influence extends to conditions such as polycystic ovary syndrome (PCOS). A study by Lindheim et al. (2017) found significant differences in the gut microbiota of women with PCOS compared to healthy controls, suggesting a potential role of the microbiome in this disorder [145]. The microbiome’s impact on insulin resistance, a key feature of PCOS, and its potential influence on androgen levels are areas of active research. Regarding pediatric endocrine disorders, such as growth hormone deficiency and early-onset puberty, Emerging research suggests that alterations in the gut microbiome during critical developmental periods can impact the hormonal regulation of growth and pubertal development. A study by Vatanen et al. (2016) on the development of the gut microbiome in infants suggests that early microbial colonization patterns may have long-term implications for endocrine health [144].

### 4.5. Bone Health

The role of the gut microbiome in bone health, an often-overlooked aspect of endocrine function, is another area of growing interest. The microbiome influences the regulation of calcium and phosphate metabolism, which are critical for bone health. A study by Schwarzer et al. (2016) showed that the gut microbiota affects the expression of genes involved in bone density and turnover, indicating a potential link between gut health and osteoporosis [146].

In conclusion, the gut microbiome’s role in endocrine disorders is multifaceted and extends to various aspects of hormonal regulation and endocrine function. From its influence on metabolic disorders like diabetes and obesity to its impact on sex hormones, adrenal health, bone metabolism, reproductive disorders, and pediatric endocrinopathies, the gut microbiome is a critical factor in endocrine health. This burgeoning field of research not only enhances our understanding of endocrine disorders but also opens new avenues for therapeutic interventions targeting the gut microbiome. As our knowledge of the gut microbiome continues to expand, it holds the promise of more personalized and effective approaches to managing a wide range of endocrine disorders.

## 5. Mechanisms by Which Microbiota Can Influence Hormone Regulation

The research into the mechanisms by which microbiota influence hormone regulation is of critical importance and scientific relevance for several reasons. First, understanding how the gut microbiota affects hormone synthesis and metabolism opens new avenues for therapeutic interventions in hormonal imbalances and related diseases.

### Gut, Hormonal Dysregulation, and Health

One of the key functions of the gut microbiota is the synthesis of essential amino acids and vitamins, such as vitamin K and certain B vitamins. These nutrients are crucial for the synthesis of steroid hormones. Clarke et al. (2014) highlight the significance of gut microbiota in the biosynthesis of these essential nutrients and their subsequent impact on host metabolism and hormonal balance [147]. This synthesis is not just limited to vitamins but extends to other compounds that play a role in hormonal regulation. For instance, certain gut bacteria are involved in the production of short-chain fatty acids (SCFAs) like butyrate, propionate, and acetate, which have been shown to influence the host’s energy metabolism and insulin sensitivity, as discussed by Canfora et al. (2015) [148].

Moreover, the microbiota can influence the activity of enzymes that metabolize hormones. For example, beta-glucuronidase, an enzyme produced by certain gut bacteria is involved in the deconjugation of estrogen. This process can lead to the reabsorption of free estrogen back into the bloodstream, thus affecting overall estrogen levels. This mechanism is elaborated by Plottel and Blaser (2011), who discuss how alterations in the microbiome can impact estrogen metabolism and potentially influence the risk of estrogen-related diseases [149]. As mentioned, the gut microbiota also plays a role in modulating stress hormones. The gut-brain axis, a bidirectional communication pathway between the gut microbiota and the central nervous system, influences the regulation of stress hormones like cortisol. Research by Foster and Neufeld (2013) demonstrates how gut bacteria can influence the hypothalamic-pituitary-adrenal (HPA) axis, thereby affecting the production and regulation of stress hormones [150]. This interaction suggests that the microbiota can indirectly influence various physiological processes modulated by stress hormones, including immune response and metabolism.

In addition to these mechanisms, the microbiota has been shown to affect insulin sensitivity and glucose metabolism, crucial aspects of hormonal regulation related to metabolic health. A study by Vrieze et al. (2012) indicates that transferring intestinal microbiota from lean donors to individuals with metabolic syndrome can lead to increased insulin sensitivity [151]. This suggests that modulation of the gut microbiota could be a potential strategy for managing metabolic disorders. Concerning this, especially because of diabetes or obesity, as mentioned before, the influence of the microbiota on appetite-regulating hormones is an area of growing interest. Gut bacteria can influence the secretion of hormones that regulate appetite, such as ghrelin and leptin. These hormones play a significant role in signaling hunger and satiety to the brain. A study by Fetissov et al. (2008) suggests that certain gut bacteria can modulate the levels of these appetite-regulating hormones, thereby influencing eating behavior and potentially contributing to conditions like obesity [152]. Furthermore, the modulation of these hormones by the gut microbiota not only affects eating behaviors but also has broader implications for energy balance and body weight regulation. This concept is further explored in the research by Le Chatelier et al. (2013), who examine the variations in gut microbiota composition in relation to body mass index and metabolic health, suggesting a significant link between microbiota diversity and metabolic disorders [153].

Additionally, the microbiota’s role in the synthesis of neurotransmitters presents another layer of complexity in its interaction with the host’s hormonal system. Certain gut bacteria can produce neurotransmitters, such as serotonin and gamma-aminobutyric acid (GABA), which are crucial for brain function and have been linked to mood regulation. This production can influence the host’s neurological and psychological health, as discussed by Strandwitz (2018) [154]. The gut-brain axis, therefore, not only involves hormonal regulation but also encompasses neural pathways, where microbiota-derived neurotransmitters play a significant role. Additionally, the impact of the microbiota on the immune system further illustrates its influence on hormonal regulation. The gut microbiota is known to modulate the immune response, which in turn can affect the production and action of various hormones. For instance, cytokines produced during immune responses can influence the function of the HPA axis, thereby affecting stress hormone levels. This intricate relationship is highlighted in the work of Rook et al. (2013), who discusses the role of microbiota in the regulation of the immune system and its subsequent impact on the endocrine system [155].

Moreover, the microbiota’s influence extends to the detoxification processes in the liver, which are crucial for hormone metabolism. The liver is a primary site for the metabolism and detoxification of hormones, and gut bacteria can produce compounds that affect liver function. This interaction is crucial for understanding conditions like estrogen dominance and hormonal imbalances. The research by Wahlström et al. (2016) provides insights into how gut microbiota influences bile acid metabolism in the liver, which is directly related to the metabolism of steroid hormones [156]. Concerning our age, the role of the microbiota in aging and the associated hormonal changes is another area of significant interest. As individuals age, changes in the composition of the gut microbiota have been observed, which could influence the aging process and the development of age-related hormonal disorders. This aspect is explored by Biagi et al. (2016), who investigate the changes in gut microbiota composition with aging and their potential implications for health and disease [157,158].

## 6. Microbiota’s Impact on Endocrine-Related Cancers

Recent literature has revealed a strong association between the microbiota and different diseases, including cancer [159]. Hence, the latest research indicates that an imbalance in the gut microbiota can initiate immune system activation and inflammation processes, directly linked to the development of cancer [160]. Among these, endocrine cancers constitute a significant area of interest due to their intricate connection with hormonal imbalances and their substantial impact on human health.

The endocrine system coordinates crucial physiological processes through hormonal signaling. Any disruption that affects this system can lead to the development of endocrine cancers. In this line, the gut microbiota plays a key role in modulating the endocrine system. Gut microorganisms actively participate in the metabolism of hormones, the synthesis of bioactive compounds, and the maintenance of host immune responses, thereby influencing endocrine homeostasis [161,162,163,164]. For example, *E. faecalis*, *E. coli*, and *B. fragilis* have been proposed as important modulators in different signaling pathways, including NF-κB, JAK1/STAT3, PI3K, and Wnt/β-catenin, by producing genotoxic compounds that affect all these routes [165,166]. In addition, microorganisms such as *P. gingivalis* or *F. nucleatum* have been related to induced chronic inflammation production as well as to oncometabolite synthesis [159]. Moreover, recent literature suggested that *F. nucleatum* was notably more prevalent in mucosal and fecal samples from individuals with colorectal cancer compared to those without the condition. These findings suggested that *F. nucleatum* could infiltrate colorectal tumors, suggesting its potential role in influencing the development of tumors [167].

### 6.1. Tumor Development

The mechanisms through which the microbiota influences endocrine cancers are complex and varied. In general, two different mechanisms have been suggested as responsible for tumor formation. Firstly, microorganisms produce metabolites, which may act as carcinogens or influence modifications in hormone levels, thereby impacting cancer development. Secondly, it has been highlighted how microbial dysbiosis may trigger chronic inflammation and disrupt the delicate balance of the endocrine system, enhancing an environment that may trigger tumor initiation and progression [168]. In this line, extensive literature has highlighted the importance of microbiota depending on tumor type, including thyroid cancer, lung cancer, pancreatic cancer, liver cancer, and colorectal cancer.

### 6.2. Dysbiosis and Types of Cancer

In terms of thyroid cancer, several authors pointed out how gut microbiote dysbiosis may compromise thyroid functioning. Hence, gut microbiote modifications, including a higher *Firmicutes* and *Bacteroidetes* ratio as well as a decreased *Butyricimonas* and *Lactobacillus* ratio, may modify short-chain fatty acids, which enhance tumor proliferation as their deficiency constitutes an oxidative environment [114].

Regarding colorectal cancer, previous researchers pointed out how some species of gut microbiota, including *E. faecalis*, *E. coli*, *B. fragilis*, *S. bovis*, *F. nucleatum*, and *H. pylori*, were capable of producing genotoxic elements such as colibactin, *B. fragilis* toxin, and typhoid toxin. These substances may be related to cancer development as they cause host DNA damage [167,169]. Thus, the signaling pathways suggested to be affected are E-cadherin/β-catenin, TLR4/MYD88/NF-κB, and SMO/RAS/p38 MAPK [166]. Moreover, it was reported by previous authors that microbiota dysbiosis products may enhance epithelial barrier impairment, triggering an inflammation environment, which consequently promotes the start and enhancement of colorectal cancer [169,170].

Concerning pancreatic cancer, recent literature proposes that gut microbiota dysbiosis may promote tumor development due to its capability of modifying the immune response within the tumor microenvironment. Hence, it has been suggested that distinct gut and intrapancreatic microorganisms assist in creating an immunosuppressive environment in pancreatic ductal adenocarcinoma within natural murine models, amplifying the advancement of cancer and fortifying resistance against immunotherapeutic interventions [171]. Thus, it may be explained due to the fact that microorganisms in pancreatic ductal adenocarcinoma activate specific TLRs in monocytes, which may be related to immune tolerance. Then, this activation may activate the NF-κB or MAPK pathway, triggering procarcinogenic effects [172].

Regarding lung cancer, recent studies showed a correlation between its presence and the gut microbiota [173,174,175]. Hence, the most likely mechanism of control could be modulating the tumoral microenvironment by modifying the regulation of B and T cells, which reach the lungs through lymphatic or hematogenous pathways and may immunomodulate different processes, regulating cell differentiation [176,177,178]. Additionally, it could also be related to inflammatory pathways, since a study conducted in mice revealed how administering a high-fiber diet to this population decreased inflammatory cell infiltration, thereby increasing defense against allergic pulmonary inflammation [179]. Again, it may be explained by the short-chain fatty acids’ antioxidant activity.

Relating to liver cancer, several authors proposed how gut dysbiosis may affect different stages of liver illness, including non-alcoholic fatty liver disease, nonalcoholic steatohepatitis, and hepatocellular carcinoma [180,181,182,183]. Hence, recent literature has proposed the potential pathways involved in liver damage, including the discharge of cancer-inducing and senescence-triggering substances by the altered microbiota, such as deoxycholic acid and bile acid. Additionally, it has also been described as a possible mechanism for the exposure of different microbe-associated molecular patterns (MAMPs), such as lipopolysaccharide, which may activate TLR4, subsequently promoting liver inflammation, fibrosis, cellular multiplication, and anti-apoptotic activation processes [183,184,185]. In addition, recent research indicated the controversial effect of short-chain fatty acids on the liver, since although they have an anti-inflammatory effect, excessive production of these components due to gut microbiota dysbiosis may favor the development of hepatocellular carcinoma through the early appearance of cholestasis and hepatocyte death [186,187,188].

### 6.3. Therapeutic Interventions

Understanding the microbiota’s role in endocrine cancers opens new targets for novel therapeutic interventions. Targeting gut microbiota modification through fecal microbiota transplantation holds promise for modulating hormonal imbalances and mitigating cancer risks, as it reduces inflammation and maintains intestinal epitelial integrity [189,190]. Additionally, personalized approaches considering an individual’s microbiome profile may revolutionize cancer treatment by optimizing therapies based on microbiota interactions by using different combinations of probiotics and prebiotics, as they reduce inflammation and carcinogenesis [191,192]. Moreover, recent studies have proposed the possibility of modifying the microbiota in order to enhance the efficacy of chemotherapy treatment and reduce its toxicity [193].

The improvement of knowledge of the intricate interplay between the microbiota and endocrine cancers highlights the significance of considering microbiota influence in understanding cancer development and progression. Further research to elucidate these complex relationships will facilitate the development of innovative preventive and therapeutic strategies, potentially revolutionizing the landscape of cancer management.

## 7. Clinical Manifestations of Microbiota-Related Endocrine Disorders

### 7.1. Overview

The gut microbiota, as previously stated in this review, is individual to each person, with its own unique composition [194] directly related to a human’s health status. Therefore, it also impacts hormone production and the endocrine system [195]. On this line, the human gut microbiota consists of different microorganisms, with bacteria (aerobes and anaerobes) being chief among them, but also including viruses, fungi, and protozoal communities [196,197,198]. Further on, microbiota composition is clearly epigenetic, as it is affected both by host genetics and lifestyle, including diet, environment, and exercise, as clear factors modulating gut microbiota composition, consequently affecting digestive tract health and hormonal and metabolic homeostasis [199,200]. Subsequently, any variation in microbial population, composition, or quantity, known as “Microbiota dysbiosis” [201,202,203], may affect the development of infectious diseases as well as metabolic and hormonal disorders such as type 2 diabetes mellitus, obesity [204], hypothyroidism, hyperthyroidism, and polycystic ovary syndrome [205]. Moreover, microbiota regulates lipids, lipopolysaccharides, and short-chain fatty acid synthesis, therefore influencing energy balance and inflammatory activity and leading to metabolic dysfunction, including insulin resistance and deficiency [195].

### 7.2. Metabolic Disorders Manifestations

Focusing on metabolic disorders, both type 1 and type 2 diabetes are affected by microbiota interactions with the immune system [206]. On this line, type 2 diabetes mellitus is characterized by insulin resistance and uncontrolled insulin hormone release combined with high glucose blood levels [207,208,209,210]. Further on, by fermenting indigestible complex carbohydrates, the intestinal microbiota generates short-chain fatty acids such as acetate and lactate [211,212], as well as propionate, which affects liver gluconeogenesis and lipogenesis, and butyrate, which is crucial for mucosal integrity and provides energy for the colon’s epithelium [213,214,215,216]. Furthermore, a more diverse microbiota results in a healthier individual. Therefore, any change in microbiota diversity and composition results in dysbiosis, which modifies intestinal permeability, fermentation, and mucosal structures, which may increase the risk of insulin resistance [153,217]. In addition, type 2 diabetes mellitus patients have presented with low-grade inflammation related to gram-negative bacteria in the gut [218,219]. Further on, the dysbiosis in type 2 diabetes is characterized by a large quantity of *Bacteriodetes* and *Escherichia coli*, as well as a low number of *Clostridium*, *Roseburia*, *and Fecalibacteria* [219]. Furthermore, obesity, another metabolic disorder, has been exponentially growing in recent years, becoming a serious health concern [220,221,222,223,224,225], normally accompanied by other problems such as cancer, atherosclerosis, and metabolic syndrome [226,227,228], and characterized by heterogeneous ethology with microbiota being highlighted [229] due to the symbiotic relationship of microbiota to energy homeostasis [222,223]. On this line, gut microbiota releases metabolites crucial to appetite control by affecting the central nervous system or by regulating hormone secretion [142], regulating adenosine monophosphate kinase (AMPK) activity in hepatic and liver cells, and preventing the extra accumulation of fatty acids in hepatic and muscular tissues [221]. Therefore, any dysbiosis in gut microbiota composition may result in an increased risk of developing obesity [230,231,232]. For example, an increased proportion of *Firmicutes*, *Proteobacteria*, *Fusobacteria*, *Firmicutes/Bacteroidetes* ratio, and *Lactobacillus* and a decrease in *Bacteroidetes*, *Faecalibacterium Prausnitzii*, *Akkermansia muciniphila*, *Methanobrevibacter smithii*, and *Bifidobacterium animalis* are related to obesity [229].

### 7.3. Hormonal Dysregulation Manifestations

Furthermore, regarding hormonal disorders, polycystic ovary syndrome is a typical and prevalent endocrine problem in women, affecting 6–26% of women worldwide [233,234]. Changes in gut microbiota composition when reaching puberty have led to the hypothesis that there is a connection between sexual hormones and intestinal microbiota [235,236]. Further on, obesity and a high-fat diet in this kind of patient cause gut microbiota dysbiosis, leading to further androgen production in the ovaries, higher immune system activation, increased inflammation, and insulin resistance affecting follicle growth, leading to polycystic ovary syndrome development [237]. Moreover, there have been reports of changes in gut microbiota in patients suffering from this disease when compared to healthy individuals, presenting increased *Shigella*, *Catenibacterium*, *Pertevolla*, and decreased *Lactobacillus*, *Akkermnesia*, and *Ruminococcaceae* [234,238,239]. While also presenting changes in hormone levels like stradiol, which are believed to produce changes in the gut microbiota [233]. Furthermore, the gut microbiota is one of the many diverse factors influencing thyroid function. When proper homeostasis is maintained in the gut microbiota, it leads to positive effects on thyroid function, leading to promoted iodine and selenium uptake in the thyroid gland; on the other hand, alterations in the gut microbiota represent a greater risk for thyroid problems [240]. On this line, it has been hypothesized that gut microbiota alterations compete with host thyroid cells for selenium, therefore producing a lack of selenium for thyroid cells and eventually altering normal thyroid function [241,242]. There are two main thyroid disorders: hyperthyroidism and hypothyroidism, which can be influenced by the gut microbiota. Hyperthyroidism is caused by an increase in T3 and T4 hormones, and hypothyroidism is caused by a reduction in thyroid hormone production all together [243,244,245]. The most common Hypothyroid disorder is known as Hashimoto thyroiditis [243,244,245]. Many factors are considered to affect the prognosis of this disease, such as female gender, pregnancy, smoking, and stress [244,246]. However, there is clear evidence of an alteration of the gut microbiota in these patients, with a decrease in *Lachnoclostridium* and *Bacteroides genera* and an increase in *Blautia* and *Romboustia* [247]. Further on, Graves’ disease is the most famous example of hyperthyroidism, accounting for 70% of all hyperthyroid patients. Gut dysbiosis is being suggested as a new hypothesis for the etiology of this disease [244,248] by a significant reduction in *Lactobacillus* and growth in the number of *Clostridum* and *Entrococcus* [205]. Thus opening a new detection and treatment approach for these diseases.

### 7.4. Osteoporosis Manifestations

Further, osteoporosis could also potentially be affected by the microbiota. On this line, osteoporosis is characterized by a decrease in bone density [249,250]. While bone density is regulated by osteoblast and osteoclast activity [251,252], an alteration in osteoclast number and quality may increase the risk of developing osteoporosis [251,253]. Moreover, there is evidence demonstrating the role of the gut microbiota in the development of osteoporosis by influencing vitamin D levels, calcium reabsorption, inflammation, immunity, and the endocrine system. This dysbiosis is characterized by a higher level of *Firmicutes*, which increases the chance of inflammation, therefore activating osteoclasts by stimulating the tumor necrosis factor alpha (TNF-α) and the interleukin-1 (IL-1) pathways [254,255,256].

After this meticulous examination of the clinical manifestations of microbiota-related endocrine disorders, we can conclude that microbiota is a crucial factor in the epidemiology and prognosis of diseases related to the endocrine system, such as metabolic and endocrine diseases. Any dysbiosis or variation in the microbiota composition may affect nutrient absorption, metabolism, and the regulating systems of various processes in the body, therefore contributing to the risk of developing this disease. As such, the maintenance of a good gut microbiota with an abundance of different species in the proportion recommended by scientific evidence is crucial to maintaining an adequate health state.

## 8. Diagnostic Tools and Biomarkers for Assessing Microbiota-Endocrine Interactions

### 8.1. Overview

The intricate microbial community within the human gut, referred to as the gut microbiota, consists of over 100 billion bacteria, archaea, viruses, parasites, and fungi. These diverse microorganisms engage in symbiotic interactions with the host, contributing to the complexity of this intricate system [257]. This complex association plays a vital role in preserving balanced homeostasis, encompassing functions such as nutrient absorption, vitamin production, immune system maturation, the preservation of epithelial mucosa integrity, and resilience against pathogens [258,259]. Due to the mutually beneficial connection between the host and gut microbiota, deviations from the typical microbiota composition, termed dysbiosis, have been identified in various human ailments [260,261]. Therefore, microbiota analysis could emerge as a potential screening tool for multiple diseases.

### 8.2. Metagenomic Sequencing

On this line, the advancement of methods for sequencing the bacterial 16s ribosomal RNA gene has facilitated the comprehensive evaluation of the overall taxonomic composition of the gut microbiome, which has significantly broadened human comprehension of the considerable variations within the population of microbiota [13]. Additionally, genes in the community of gut microorganisms are involved in functions including the development and stimulation of the immune system, digestion, and degradation in the digestive tract [262,263]. In recent times, metagenomic sequencing (MGS) has emerged as an effective method for researching the microbiome. MGS can identify bacteria, viruses, protozoa, and fungi; the examination of bacterial genes and the prediction of biological pathways will be achievable through this innovative technology [264]. Furthermore, growing attention has been directed towards investigating the influence of gut microbiota on human health, specifically concerning metabolic disorders like obesity, type 2 diabetes, and dyslipidemia, which elevate the susceptibility to cardiovascular events. It is observed that the distinct signatures of gut microbiota are highly individualized, and contingent on host genetics [195].

Further on, MGS enables the identification of specific genes and functional pathways by deciphering the entire genomic makeup of microbial communities, enabling the simultaneous analysis of bacteria, archaea, viruses, parasites, and fungi [265]. Standing at the forefront of diagnostic tools, offering a holistic view of the genetic content of the gut microbiome, and allowing researchers to understand how microbial genes contribute to endocrine regulation, providing insights into potential targets for therapeutic interventions [266]. In addition, it allows researchers to unravel the intricate genetic makeup of microbial communities residing in the human gastrointestinal tract [267]. Furthermore, metagenomic sequencing goes beyond taxonomic classification, facilitating functional profiling and the discovery of novel microbial genes involved in host-microbe interactions or crucial roles in maintaining homeostasis [268,269]. It unveils the dynamic nature of the gut microbiota, offering insights into how microbial communities evolve over time and in response to various factors [270,271]. Longitudinal studies using this technique enable the tracking of microbial shifts associated with dietary changes, antibiotic use, or the onset of diseases [272,273,274]. This holistic approach positions metagenomic sequencing as an invaluable tool in advancing our understanding of microbiota-endocrine interactions, with diagnostic and therapeutic implications [275]. Its ability to identify specific microbial signatures associated with health or disease states holds promise for personalized interventions, shaping the future of precision medicine and personalized healthcare by harnessing the power of the gut microbiome [276]. Additionally, another tool used for gut microbiota analysis would be 16S ribosomal RNA gene sequencing [277]. This method enables high-resolution taxonomic classification, offering insights into the diversity and abundance of bacterial communities [277]. This technique can associate specific bacterial taxa with health and disease states, including obesity and inflammatory bowel diseases [278]. Its integration with metagenomic approaches offers a comprehensive view of both microbial taxonomy and functional potential [265], bridging the gap between microbial composition and the roles these microorganisms play in host physiology [279]. Therefore, as technology advances, ongoing improvements in sequencing and bioinformatics tools will enhance the precision of gut microbiota analysis, making 16S rRNA gene sequencing an invaluable asset in unraveling the complex microbial communities within the gastrointestinal tract [280].

### 8.3. Metabolics and Proteomics

Furthermore, intricate exploration of gut microbiota metabolomics and proteomics emerge as powerful tools by providing a profound understanding of the functional dynamics within microbial communities [281]. Metabolism studies metabolites (small molecules reflective of ongoing metabolic processes within the gut ecosystem), such as short-chain fatty acids and amino acid derivatives, and proteomics scrutinizes the entirety of protein production [282,283]. Both approaches provide a holistic view of gut microbiota function by unveiling insights into biological processes, metabolic pathways, and enzymatic activities [284], enhancing our understanding of how microorganisms influence nutrient metabolism and energy production [285]. Beyond mere taxonomic classification, proteomics and metabolomics delve into the intricate molecular interactions between the microbiota and the host, elucidating the roles of microbial proteins in adhesion, host immune modulation, and the synthesis of bioactive molecules [286]. Moreover, metabolomics serves as a crucial point in biomarker discovery for microbiota-related disorders, unveiling distinct metabolic profiles associated with conditions like inflammatory bowel diseases and obesity [287]. While proteomics sheds light on the dietary composition, influencing the expression of microbial proteins and impacting nutrient metabolism and bioactive molecule synthesis [288], their integration with other omics approaches, including metagenomics and transcriptomics, enables a comprehensive understanding of the interplay between microbial genes, gene expression, and metabolite production, bridging the gap between microbial composition and its impact on host metabolism [289]. Furthermore, these methods unravel the functional dynamics of the gut microbiota, contributing not only to our understanding of host-microbe interactions but also to biomarker discovery for microbiota-related disorders [290].

Moreover, the holistic evaluation of microbiota-endocrine interactions involves integrating diverse biomarkers, each providing unique insights into the intricate relationship between gut microbiota and endocrine regulation [291]. Short-chain fatty acids (SCFAs), including acetate, propionate, and butyrate, emerge as crucial microbial metabolites with direct implications for endocrine function, and monitoring their levels in biological samples serves as a valuable biomarker, indicating the metabolic activity of the gut microbiota and potential disruptions in the microbiota-endocrine axis [292]. Additionally, biomarkers associated with the abundance or scarcity of specific microbial taxa offer nuanced insights into the microbial landscape influencing endocrine health [293]. Monitoring shifts in microbial composition, particularly concerning key taxa, serves as a diagnostic indicator for conditions linked to dysbiosis, encompassing metabolic and hormonal disorders [294]. Further insights are gleaned from measuring hormone levels (e.g., insulin, ghrelin, and leptin) and pro-inflammatory cytokines, providing direct information on the impact of the gut microbiota on endocrine function [295]. Alterations in these biomarkers may signify dysregulation in metabolic and immune responses, offering valuable diagnostic information [296]. Additionally, exploring specific genetic markers within the microbial genome adds another layer of complexity to endocrine-related diagnostics, enabling a more targeted approach and potentially paving the way for personalized interventions [297]. This integrative use of diverse biomarkers enhances the precision and comprehensiveness of microbiota-endocrine assessments, fostering a deeper understanding of this intricate interplay for both diagnostic and therapeutic purposes [298,299,300].

In conclusion, advances in sequencing technologies, such as 16S ribosomal RNA gene sequencing and MGS, have significantly expanded our understanding of the gut microbiome’s taxonomic composition and functional potential, allowing a comprehensive evaluation of the genetic content of the gut microbiome. Moreover, the integration of diverse biomarkers, including those from metabolomics and proteomics, provides a holistic view of the functional dynamics within the gut microbiota.

## 9. Current Therapeutic Approaches Targeting Microbiota-Endocrine Crosstalk

### 9.1. Overview

Understanding the interaction between microbiota and the endocrine system has led to significant interest in therapeutic approaches aimed at modulating this interaction for treating various conditions. In this line, previous literature has shown that this microbiota significantly influences the endocrine system, participating in the regulation of metabolism, immune function, and hormonal synthesis. Hence, dysbiosis has been associated with endocrine diseases such as type 2 diabetes, obesity, and inflammatory bowel disease [301,302,303,304].

### 9.2. Pre and Probiotics

One emerging therapeutic approach involves the use of prebiotics and probiotics in order to modulate the microbiota and improve endocrine health. Probiotics, beneficial bacterial strains, and prebiotics substrates that promote their growth, have shown beneficial effects on hormonal and metabolic regulation [305,306,307]. Moreover, the use of symbiotics, products that combine both substances, prebiotics, and probiotics, is expected to yield a more potent effect compared to the individual efficacy of either the probiotic or prebiotic when used separately [308]. Clinical studies have demonstrated that supplementation with certain probiotic strains can improve diabetes and different metabolic diseases, such as obesity [307,309]. Additionally, recent literature proposes the use of fecal transplants in order to restore bacterial flora with the aim of improving the patient’s microbiota and thus improving or preventing different pathologies such as diabetes mellitus, metabolic syndrome, or polycystic ovary syndrome [310,311]. Finally, modifications in diet patterns have also been pointed out as a useful tool that may improve the microbiota [312].

More specifically, regarding probiotic utilization, it has been proposed that administering certain bacterial strains, such as *Bifidobacterium* and *Lactobacillus*, has shown encouraging results in regulating blood glucose and reducing inflammation, which could be relevant for type 1 diabetes mellitus treatment. Hence, this study suggested that a significant reduction in fasting blood glucose levels exists in those patients treated with probiotics, as well as a reduction in IL-8, IL-17, and TNF-α, revealing the important role in the regulation of immune cytokines that probiotics may have [313]. Similar results were found in rats, as the administration of a multi-strain probiotic supplement that included *Lactobaccilus salivarius*, *Lactobaccilus johnsonii*, *Lactobaccilus reuteri*, and *Bifidobacterium animalis* reduced TNF-α, IL-6, and IL-1β levels in streptozotocin-induced diabetic rats, showing the protective effect of probiotics in β-cells, stabilizing glycemic levels, and decreasing inflammation [314]. Moreover, recent researchers pointed out how supplementation with certain probiotic strains may improve insulin sensitivity in insulin-resistant patients and modulate the secretion of GLP-1 and GLP-2. These peptides contribute to reducing low-grade inflammation linked to diabetes, lowering insulin resistance, subsequently reducing ß-cell toxicity, and enhancing glycemic control. Moreover, GLP 1 and GLP 2 play a role in diminishing hunger and promoting satiety, leading to a reduction in energy intake. As a result, these effects contribute to improved glycemic control [315].

### 9.3. Diet

Regarding diet modifications, it has been described in previous literature how the consumption of diets rich in fiber, polyphenols, and omega-3 fatty acids has been shown to positively influence microbiota composition and, consequently, endocrine activity. These dietary components not only promote the growth of beneficial bacteria but also possess anti-inflammatory and antioxidant effects, impacting hormonal and metabolic regulation. In this line, previous researchers pointed out the key role that whole-grain consumption may have in preventing type 2 diabetes [316]. Moreover, a specific type of macrobiotic diet, the Ma-pi diet, has been described as useful for reducing type 2 diabetes mellitus. This activity may be explained by the fact that this diet comprises complex carbohydrates, beans, fermented foods, sea salt, and green tea, products that have been described by their capability of promoting gut microbiota diversity and increasing the growth of beneficial bacteria, such as *Faecalibacterium*, *Bacterioides*, and *Akkermansia*, highlighted as important short-chain fatty acids and mucus productors [317,318]. According to these findings, it could be considered that dietary fiber plays a crucial role in microbiota modulation, as it enriches microbiota composed by bacteria that produce short-chain fatty acids, which stimulate intestinal cells to release GLP-1 and the peptide YY [319]. As a consequence, glucose may be reduced in these patients due to the activity of both hormones suppressing hunger, increasing insulin release, and diminishing glucagon production. Regarding polyphenols, it has been described by previous researchers how green tea polyphenols inhibited the proliferation of multiple strains of bacterial pathogens, including *L. monocytogenes*, *P. aeruginosa*, *S. aureus*, and *B. cereus* [320]. Moreover, it has also been described how quercetin and chlorogenic acid, two polyphenols present in blueberries, may decrease growth in different strains of bacterial pathogens, including *Listeria monocytogenes*, *Salmonella enteritidis*, *Helicobacter pylori*, and *Bacillus cereus* [321,322]. In addition, research literature has proposed the fascinating role that microbial metabolism may have, since urolithin A, a microbial substance that comes from the metabolism of polyphenols from blueberries and pomegranate fruits, has presented anti-inflammatory properties as well as contributing to improving barrier integrity [323]. Hence, it could be considered how polyphenol intake may modulate microbiota composition, offering health benefits related to their antioxidant properties [324,325,326,327]. Finally, regarding omega-3 fatty acids, recent research proposed the important role eicosapentaenoic acid and docosahexaenoic acid may have in the gut microbiota, as it was described how their supplementation may contribute to an increase in *Bifidobacterium* and *Oscillospira* genera, linked to a reduction of *Coprococcus* and *Faecalibacterium*. Additionally, a raise in *Lachnospira* and *Roseburia* genera was found. Thus, this research highlighted the increased presence of bacterial genera responsible for producing butyrate (a short-chain fatty acid) following supplementation with omega-3 PUFA [328]. Furthermore, it has been described as the potential effect of omega-3 fatty acids modulating the microbiota and having a positive effect on liver disease. Thus, they reduce lipopolysaccharide and inflammatory mediators such as TNF-α, Il-6, IL-18, and NF-κB, reducing nonalcoholic steatohepatitis progression [329].

### 9.4. Fecal Transplant

Finally, recent literature has proposed the use of fecal transplants as a useful tool that may be helpful in different diseases, including diabetes and obesity. Thus, it has been described by recent authors how fecal microbiota transplantation from healthy donors effectively halted, or at the very least notably decelerated, the progression of type 1 diabetes in patients who had recently developed the condition (within less than six weeks) [330]. Moreover, interesting results were found by the latest researchers, as they have noticed how important and precise the metabolites produced by microbiota are, as protection against diabetes was actually observed following the transplantation of microbiota influenced by a diet rich in acetate rather than butyrate [331]. Similar results were found in obesity in a study conducted in mice. Thus, it revealed how transplantations of adult human fecal microbiota into germ-free (GF) mice may modulate adiposity. Then, these humanized mice were divided into groups fed with a low-fat or high-fat diet. GF mice were colonized with gut microbiota from humanized donors who had been on a low-fat or high-fat diet. After this moment, all mice were maintained on a low-fat diet. Mice colonized with microbiota from donors on a high-fat diet exhibited notably higher adiposity compared to those colonized with microbiota from donors on a low-fat diet [332]. Then, these results showed that fecal transplants have further illustrated the potential causal role of gut microbiota in the onset of metabolic disorders linked to obesity.

Thus, according to these results, it could be considered how the interaction between the microbiota and the endocrine system has opened new therapeutic perspectives for treating various diseases. From the use of probiotics and prebiotics to dietary modulation and fecal transplant, current approaches aim to restore microbial balance and improve endocrine health. However, further research is needed to fully understand the underlying mechanisms and develop more specific and effective therapies.

## 10. Challenges and Limitations in Studying Microbiota Endocrine Interactions

Studying the interactions between the microbiota and the endocrine system presents several challenges and limitations that impact our understanding and the development of therapeutic interventions. A key challenge is the complexity of the microbiota itself, which consists of an immense diversity of microorganisms, each potentially playing a unique role in endocrine regulation. This complexity makes it difficult to identify specific causal relationships and mechanisms of action. Another significant limitation is the variability of the microbiota among individuals, influenced by factors such as genetics, diet, environment, and lifestyle. This variability can lead to inconsistent results across studies, complicating the identification of universal patterns or treatment approaches.

Furthermore, the majority of current research relies on correlational studies, which do not establish causation. Experimental studies, particularly on human subjects, are scarce due to ethical and practical constraints. This limits our ability to determine the direct effects of microbiota manipulation on endocrine-related conditions. The dynamic nature of the microbiota, which can change rapidly in response to various factors, poses an additional challenge for researchers. It necessitates the use of longitudinal studies to understand how these changes over time affect endocrine health.

Finally, there is a need for more advanced analytical tools and methods to study microbiota-endocrine interactions. Current methodologies may not be sufficiently sensitive or specific to detect subtle but potentially significant changes in the microbiota or its metabolic products. While the field of microbiota-endocrine research is promising, it is fraught with challenges that require innovative solutions and a multidisciplinary approach to overcome. Addressing these limitations is crucial for advancing our understanding and developing effective microbiota-based therapies for endocrine disorders.

In summary:

– Microbiota Complexity: The microbiota’s immense diversity poses a significant challenge.
– Individual Variability: Microbiota variability among individuals is influenced by genetics, diet, environment, and lifestyle.
– Correlational Studies Dominance: The majority of research relies on correlational studies, lacking causal establishment.
– Dynamic Nature of Microbiota: The microbiota’s dynamic nature is changing rapidly in response to various factors.
– Analytical Tools and Methodology: Need for more advanced analytical tools and methods
– Promising yet Challenging Research Field: Despite the promising nature of microbiota-endocrine research

## 11. Future Directions for Research in this Field

As we venture into the uncharted territories of microbiota-endocrine system research, it becomes crucial to outline the prospective directions this field might take. Recent advancements have illuminated the intricate connections between our gut microbiota and the endocrine system, offering novel insights into their interplay. This introductory section aims to set the stage for discussing potential avenues in microbiota-related research, particularly focusing on their impact on endocrine health and the development of innovative therapeutic strategies. Exploring the modulation of gut microbiota in the context of broader endocrine-related conditions presents a vital research trajectory. The significant work of Ley et al. (2006) [230] and Turnbaugh et al. (2007) [232] lays a foundation for investigating how alterations in microbiota impact the hypothalamic-pituitary-adrenal (HPA) axis, a key player in stress response and metabolic processes. This line of research could unveil novel treatment avenues for stress-related and metabolic endocrine disorders, enhancing our understanding of the complex interactions between the microbiota and the endocrine system.

### 11.1. Microbiota-Thyroid Research

Given the thyroid’s crucial role in metabolism, research could investigate the connections between gut health and thyroid disorders, as suggested by some studies [333,334]. This area, though not extensively explored, could provide valuable insights into the microbiota-thyroid axis. Employing advanced analytical techniques such as metabolomics and bioinformatics could help unravel the complex interactions and lead to innovative therapeutic strategies for thyroid disorders, thereby broadening our understanding of the microbiota-endocrine relationship. Expanding further, research should also consider the intricate relationship between gut microbiota and autoimmune thyroid disorders, such as Hashimoto’s thyroiditis and Graves’ disease. Although initial studies indicate a potential link, more comprehensive research is needed to elucidate these connections [247,335]. Additionally, exploring how diet influences the gut microbiota and consequently, thyroid function could open new non-pharmacological management strategies for thyroid disorders. This approach aligns with the emerging interest in personalized medicine and lifestyle interventions in endocrine health.

### 11.2. Focus on the Interrelationship between Gut Bacteria and Hormone Control

Expanding on the microbiota’s intricate influence on various endocrine systems, including the adrenal and pituitary glands, presents a fascinating avenue for future investigation. A growing body of research has begun to shed light on the interconnectedness of gut microbiota with hormone regulation. For instance, Li et al. (2019) conducted a comprehensive study that delved into the relationship between gut microbiota composition and cortisol metabolism, revealing how the microbiota may play a role in modulating stress responses and cortisol levels [336]. This finding not only underscores the gut’s potential impact on the adrenal system but also suggests a connection between the gut microbiome and conditions related to stress and hormonal imbalance. Moreover, as we consider the broader endocrine landscape, emerging studies like those by Torres et al. (2018) have explored the intriguing interplay between gut microbiota and sex hormones [337]. Their research demonstrated that the gut microbial diversity in women with polycystic ovary syndrome (PCOS) correlated with hyperandrogenism, providing crucial insights into the potential role of the microbiota in conditions affecting reproductive health [337]. This discovery opens up new possibilities for understanding and managing PCOS and other hormonal imbalances that significantly impact women’s health.

### 11.3. Gut Microbes and Endocrine System Connections

Expanding our exploration of the gut microbiota’s influence on various endocrine systems, it becomes evident that a comprehensive investigation should consider the collective impact of gut microbes on the entire endocrine network. The intricate crosstalk between different hormonal pathways and their interconnected regulation is a fascinating area of study. Research in this direction could unravel the complexity of how the gut microbiota influences multiple endocrine axes simultaneously, potentially contributing to the development of multi-system endocrine disorders. A notable study by Zhang et al. (2021) sheds light on the interplay between gut microbiota and the endocrine system, emphasizing the need to view endocrine health as an integrated network rather than isolated hormonal pathways [207]. Additionally, it provided insights into how dysbiosis of the gut microbiome may disrupt the coordinated regulation of various endocrine functions, leading to a cascade of interconnected health issues [338]. Furthermore, recent research by Chen et al. (2022) delves into the dynamic interactions between gut microbiota and different hormones, emphasizing the potential implications for multi-system endocrine disorders and metabolic health [339].

### 11.4. Future Research Emphasis

We present future directions for research that promise to expand our understanding of the human body’s complex systems. This section introduces three critical areas of focus:

– Microbiota-Thyroid Axis: A New Frontier: The connections between gut health and thyroid disorders, emphasizing the potential of advanced analytical techniques like metabolomics and bioinformatics.
– Interconnectedness of Gut Microbiota and Hormone Control: An exploration of the interplay between gut microbiota and sex hormones offers insights into reproductive health with potential health implications.
– Comprehensive Investigation of the Microbiota-Endocrine Network: Research in this direction could unravel the complexity of how the gut microbiota influences multiple endocrine axes simultaneously.

## 12. Conclusions

This review underscores the profound and intricate relationship between the human microbiota and endocrine-related diseases. Our exploration reveals that microbiota composition and functionality are pivotal in influencing endocrine health, opening avenues for innovative therapeutic strategies. Despite the complexities and challenges inherent in this field, the potential of microbiota-based interventions for managing and improving endocrine disorders is promising. However, the necessity for more comprehensive, robust, and longitudinal studies is clear to validate these approaches and deepen our understanding.

Improving the lives of patients through a comprehensive approach to microbiota management involves addressing key topics such as the impact of microbiota on hormone regulation, its role in endocrine pathologies, and the promising avenues of microbiota modulation through diet, probiotics, prebiotics, and fecal microbiota transplantation (FMT). Here is a breakdown of how each aspect contributes to enhancing the well-being of patients:

1. Understanding Microbiota’s Impact on Hormone Regulation:
  – Patient Education: Providing patients with detailed information about how the microbiota influences hormone regulation helps them understand the interconnected nature of their body systems.
  – Behavioral Changes: Encouraging patients to adopt lifestyle changes that promote healthy microbiota, such as a balanced diet, regular exercise, and stress management.
2. Role of Microbiota in Endocrine Pathologies:
  – Early Detection and Monitoring: Emphasizing regular check-ups and tests to detect any alterations in the microbiota associated with endocrine pathologies, enabling early intervention.
  – Tailored Treatment Plans: Developing personalized treatment plans that consider the patient’s microbiota profile alongside traditional medical approaches for better outcomes.
3. Microbiota Modulation Through Diet:
  – Nutritional Counseling: Providing personalized nutritional counseling based on the patient’s microbiota composition to optimize their diet for improved endocrine health.
  – Incorporating Probiotic-Rich Foods: Encouraging the inclusion of probiotic-rich foods, such as yogurt and fermented products, to naturally support a healthy microbiota.
4. Probiotics and Prebiotics:
  – Supplementation Strategies: Recommending specific probiotic and prebiotic supplements tailored to the patient’s microbiota needs, potentially improving microbial diversity and function.
  – Educating Sources: Informing patients about the natural sources of probiotics and prebiotics in food enables them to make informed choices for their gut health.
5. Fecal Microbiota Transplantation (FMT):
  – Consideration in Treatment Plans: Evaluating the potential benefits of FMT for specific endocrine conditions and incorporating it into treatment plans when deemed appropriate.
  – Ensuring Safety and Regulation: Ensuring that FMT procedures adhere to safety standards and regulatory guidelines to minimize risks and optimize therapeutic outcomes.
6. Holistic Patient-Centric Approach:
  – Integration with Conventional Treatments: Integrating microbiota-focused interventions with conventional medical treatments to provide a comprehensive and holistic approach.
  – Patient Empowerment: Empowering patients to actively participate in their healthcare by making informed decisions about their diet, lifestyle, and treatment choices based on their microbiota profile.

By focusing on these aspects, healthcare providers can contribute to an improved quality of life for patients by leveraging the intricate relationship between microbiota and endocrine health. This patient-centered approach aims to enhance overall well-being and potentially offer more effective and personalized solutions for managing endocrine-related conditions.
